# Fabrication of silane-grafted graphene oxide and its effect on the structural, thermal, mechanical, and hysteretic behavior of polyurethane

**DOI:** 10.1038/s41598-020-76153-8

**Published:** 2020-11-05

**Authors:** Joo Hyung Lee, Seong Hun Kim

**Affiliations:** Department of Organic and Nano Engineering, Hanyang University, Seoul, Republic of Korea

**Keywords:** Composites, Nanoparticles

## Abstract

Incorporation of nanofillers into polyurethane (PU) is a promising technique for enhancing its thermal and mechanical properties. Silane grafting has been used as a surface treatment for the functionalization of graphene oxide (GO) with numerous reactive sites dispersed on its basal plane and edge. In this study, amine-grafted GO was prepared using silanization of GO with (3-aminopropyl)triethoxysilane. The functionalized graphene oxide (fGO) was characterized by Fourier transform infrared spectroscopy (FT-IR) and X-ray photoelectron spectroscopy. Next, it was introduced in PU fabricated using polycaprolactone diol, castor oil, and hexamethylene diisocyanate. The fGO–PU nanocomposites were in turn characterized by FT-IR, X-ray diffraction, scanning electron microscopy, differential scanning calorimetry, thermogravimetric analysis, and a universal testing machine. The results obtained from these analyses showed changes in structural thermal properties, as well as improved thermal stability and mechanical properties because of the strong interfacial adhesion between the fGO and the PU matrix.

## Introduction

Vegetable oil-based hyperbranched polyurethane (PU) is one of the most important classes of polymer because it is an eco-friendly material that responds to environmental concerns such as plastic garbage pollution, depletion of fossil oils, CO_2_ emissions, and global warming^1−5^. Plant oils have received much attention as petroleum replacements in manufacturing commercial polyols used to produce PU because they are abundant and easy to extract from biorenewable resources such as castor, soybean, canola, sunflower, grapeseed, and palm^6−13^. Vegetable oils also have a great potential to apply on biopolyurethanes due to its unique characteristics originated from triglyceride structure combined three fatty acids to glycerol. Thus, many studies have focused on biobased hyperbranched PUs, which have a nonentangled three-dimensional (3D) structure, low melting point, and solution viscosity, high reactivity, etc.^14−16^.

Graphene has received much attention in the field of graphene/polymer nanocomposites due to its unique characteristics such as excellent stiffness, strength, and thermal and electrical conductivities^17−20^. There are two main types of graphene structure available as a filler; one is graphene oxide (GO) and the other is its reduced form. Compared with reduced GO, which has few oxygen functionalities, GO possesses various oxygen groups, including epoxide, the hydroxyl group, and the carbonyl group on the basal planes and along the edge. Although the exact structure of GO is still a matter of debate, the abundant functional groups that are dispersed heterogeneously on the GO surface provide the advantage of producing various GO derivatives. During the last decade, numerous surface treatments and functionalization methods have been reported for enhancing the dispersibility and interfacial adhesion of GO in a polymer matrix. Among the various surface treatment techniques, silane coupling agents have been applied extensively to impart distinctive characteristics to GO^21^. The silane coupling agents are compounds with molecules containing functional groups that bond with both organic and inorganic materials. Reactive groups that form chemical bonds with organic materials such as polymers exhibit different properties depending on their type. These groups include the vinyl, epoxy, amino, and mercapto groups, etc.^22^. Their resulting properties constitute improvements in dispersibility, water resistance, interfacial adhesion, water resistance, and even flame retardancy^23−26^. In the GO/polymer nanocomposite, GOs modified with amino groups are often used. Lee and co-workers have reported carbon fiber/epoxy composites reinforced with four silane–fGOs that show a drastic improvement in bonding strength; in particular, GO modified with (3-aminopropyl)triethoxysilane (APTES) or (3-aminopropyl)trimethoxysilane (APTMS) showed up to 53% enhancement^27^. Ma et al*.* have reported the application of APTES-functionalized graphene as a reinforcement in PU^28^. They performed a reduction of APTES-grafted GO using the hydrazine hydrate reaction and confirmed its boosting effects on the mechanical properties and thermal conductivity in a PU matrix. Because of the presence of a number of functional groups on its surface, GO can take part in the PU reaction, which is synthesized by the reaction between isocyanate and the hydroxyl groups. Moreover, the NH_2_ amine groups on the amino-fGO can also participate in the PU reaction, which forms a urea linkage or greatly affects hydrogen bonding in PU. The formed urea linkage between NH_2_ amine group and urethane bonds could improve the mechanical properties of the PU by enhancing the interfacial adhesion between reinforcements and PU matrix. So far, there has been no research on these phenomena. In this study, we investigated the effects of amino-grafted GO in a PU matrix on the structural, thermal, and mechanical properties of GO functionalized with APTES. The fGO was prepared and characterized using Fourier transform infrared spectroscopy (FT-IR) and X-ray photoelectron spectroscopy (XPS). The fGO was introduced simultaneously in the synthesis process of bio-PU using castor oil with different loading level of fGO. The fGO–PU nanocomposites were characterized in terms of chemical structural analysis, crystallinity, morphology, thermal, and mechanical properties.

## Experimental

### Materials

Graphite powder (300 mesh), potassium permanganate (KMnO_4_), dibutyltin dilaurate (DBTDL) were obtained from Sigma-Aldrich, USA. APTES was purchased from Alfa Aesar, USA. Castor oil (CO) was obtained from Yakuri Pure Chemical Co., Ltd, Japan. Polycaprolactone diol (PCL-diol), initiated with neopentyl glycol (CAPA 2200A, molecular weight, MW, 2,000) was purchased from Perstorp Chemicals, Sweden. Hexamethylene diisocyanate (HDI) was purchased from Wako Pure Chemicals. Sulfuric acid (98%, H_2_SO_4_), dichloromethane (DCM), *N,N*-dimethylformamide (DMF), magnesium sulfate anhydrous (MgSO_4_), and sodium chloride (NaCl) were supplied from Daejung Chemical Co., Korea. All chemicals were used without any further purification. GO was prepared based on the modified Hummer method with a slight modification for less defective GO^29^. Briefly, 5 g of graphite was added to 115 mL of H_2_SO_4_ and stirred for 1 h in an ice-water bath. After that, 15 g of KMnO_4_ was added and the temperature was kept constant at 40 °C for 3 h, followed by 230 mL dropwise addition of deionized water to prevent a rapid increase in temperature. The temperature was increased slowly to 90 °C, and the mixture was stirred for another 20 min followed by addition of 115 mL deionized water. The product was filtered and washed several times and dried in a vacuum oven at 50 °C.

### Preparation of functionalized fGO

**Scheme 1:**
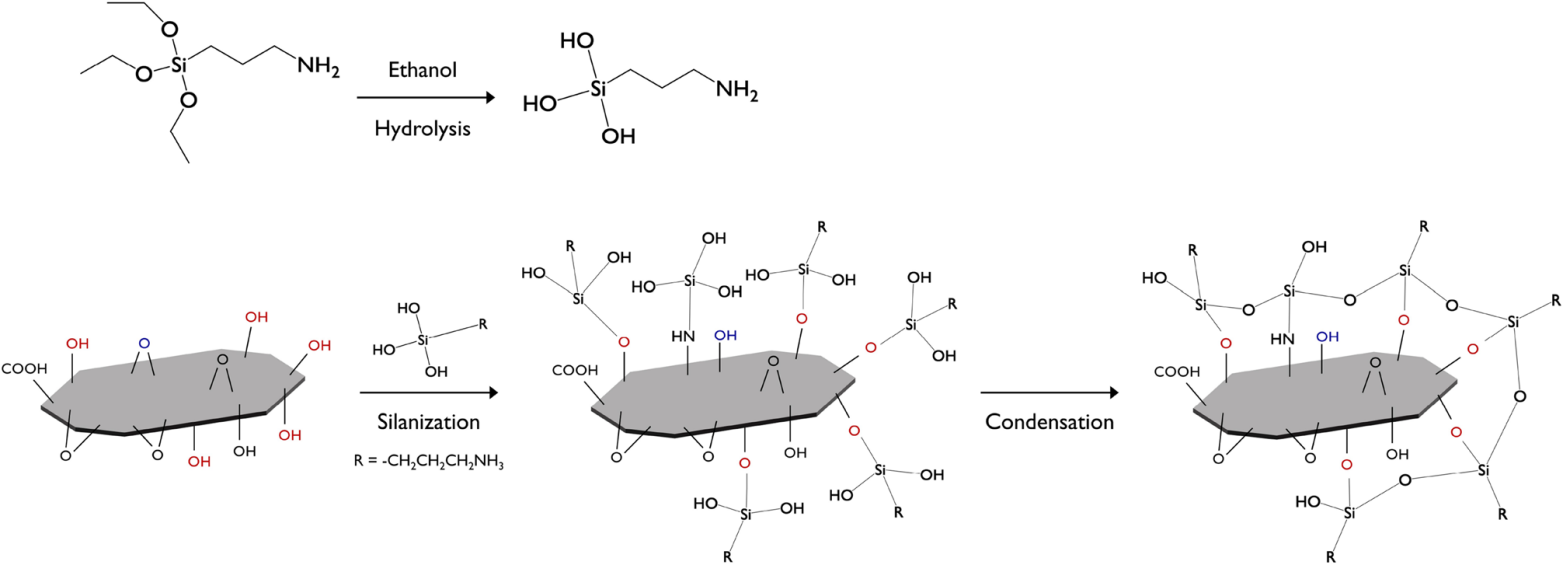
Preparation of fGO–PU nanocomposites.

0.3 g of GO was dispersed in 270 g of anhydrous ethyl alcohol through ultrasonication for 1 h; 6 g of APTES was added and the mixture was stirred vigorously at a temperature of 78 °C for 18 h. The mixture was filtered and washed several times with deionized water and ethanol. Finally, APTES-treated GO was obtained after vacuum drying at 50 °C for 12 h. The scheme of the reaction is depicted in Scheme 1.

### Preparation of fGO–PU nanocomposites

**Scheme 2:**
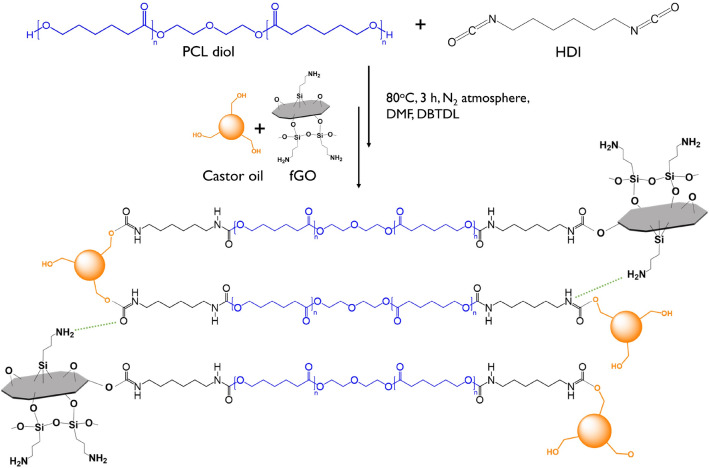
Surface modification of GO with APTES.

Before preparation of the fGO–PU nanocomposites, the CO was dried at 80 °C for 10 h in vacuo. The fGO–PU nanocomposites were prepared in a two-step polymerization process as shown in Scheme 2. In the first step, the NCO-terminated PU prepolymer was obtained by reaction of PCL-diol and HDI with a molar ratio of 1:4. The reactants were dissolved in DMF at a concentration of 10 wt% and stirred at 80 °C for 2 h in a N_2_ atmosphere with a few drops of DBTDL as the catalyst. After that, another mixture, prepared by dissolving the desired amount of CO and fGO in DMF, was added dropwise and stirred at 80 °C for 3 h. No additional catalyst was added and a N_2_ atmosphere was maintained until the reaction was completed. The solutions were cast into a PTFE-coated mold and degassed in vacuo at room temperature. The solvent was allowed to evaporate at 80 °C in a convection oven. The final ratio of NCO:OH of the fGO–PU nanocomposites was 2:1 and the fGO contents were 0, 0.05, 0.10, and 0.20 wt%. The samples were coded as fGO–PU 0, fGO–PU 0.05, fGO–PU 0.10, and fGO–PU 0.20, respectively. The detailed compositions of the fGO–PU nanocomposites are listed in Table 1.

**Table 1:** Composition of the PU obtained.

Sample	Functionality ratio of PCL-diol/HDI/CO	Total NCO/OH ratio	fGO content (wt%)	Hard segment (HS) content^*a*^	
				Weight (%)	Mol (%)
fGO–PU 0	1:4:1	2	0	40.6	82.6
fGO–PU 0.05	1:4:1	2	0.05	40.6	82.6
fGO–PU 0.10	1:4:1	2	0.10	40.6	82.6
fGO–PU 0.20	1:4:1	2	0.20	40.6	82.6

^a^Hard segment content was calculated using the equation below.

HS content = $$\frac{Weight or moles of (HDI+Crosslinker)}{Weight or moles of (HDI+Crosslinker+PCL-diol)}\times 100.$$

### Characterization

**Figure 1:**
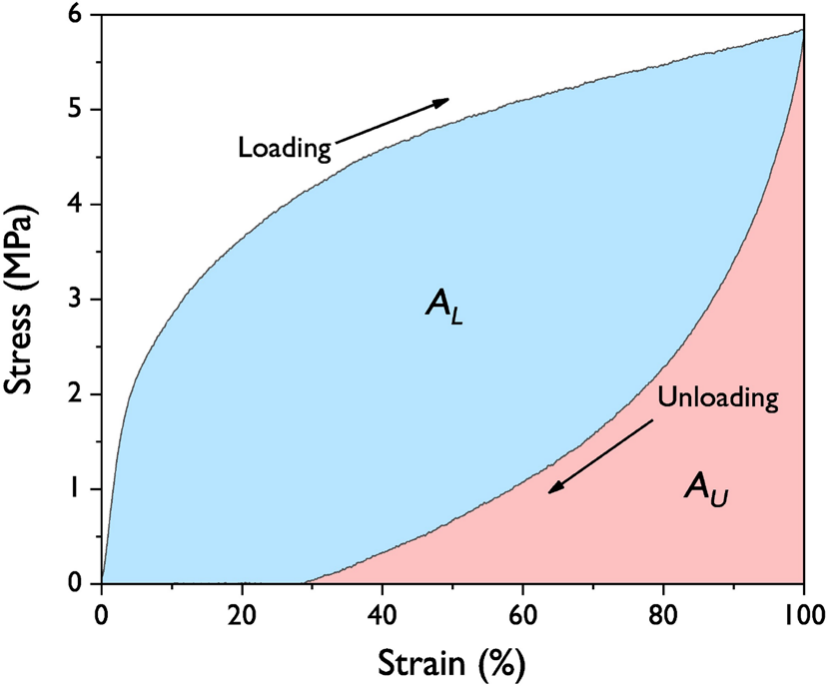
Schematic of the mechanical hysteresis quantification using the areas in the stress–strain curve.

An FT-IR (Nicolet 760 MAGNa-IR spectrometer) was used to determine the structure of the GO, fGO, and fGO–PU nanocomposites. XPS was performed using K-Alpha Plus (Thermo Fisher Scientific) with an Al K*α* X-ray radiation and the energy step size was 1.0 eV and 0.1 eV for the survey scan and narrow scan, respectively. Wide-angle X-ray diffraction (XRD) patterns were collected using an X-ray diffractometer (Rigaku, SmartLab) and diffractograms were scanned in a 2*θ* range of 5‒70° at a scan rate of 3° min^−1^. The morphologies of the fGO and fGO**–**PU nanocomposites were examined using JEOL JSM-6340F and S8010 (Hitachi Co., Japan) field emission scanning electron microscopy (FE-SEM) respectively. Thermogravimetric analysis (TGA) of the fGO–PU nanocomposite was performed under N_2_ purge from 30 to 800 °C at 20 °C min^−1^. The thermal behavior of the nanocomposites was investigated by differential scanning calorimetry (DSC, TA Instrument, DSC Q20). All samples were heated from − 80 to 160 °C at 20 °C min^−1^ under N_2_ atmosphere. Samples were held at 160 °C for 3 min and quenched to − 80 °C, and then reheated to 160 °C at the same rate. From the obtained DSC curve, thermal properties such as transition temperature (*T*_*g*_), melting temperature (*T*_*m*_), and enthalpy of melting (Δ*H*_*m*_) of the nanocomposites were calculated. The tensile properties of the fGO–PU nanocomposites were measured at room temperature using an AND MCT-1150 universal testing machine to evaluate the tensile and cyclic recovery properties. Testing specimens were prepared in the dimensions of 63 × 3 × 0.3 mm^3^ according to the ASTM D638 standard. The gauge length and the crosshead speed were set to 25 mm and 20 mm min^−1^, respectively. The cyclic recovery test was performed five times up to a strain of 100%. To quantify the mechanical hysteresis (*H*_*M*_) for the fGO–PU nanocomposites, according to Fig. 1, *H*_*M*_ was calculated using the equation below from the difference in the area under the loading and unloading curves of the cyclic recovery test^30^.

$${H}_{M}=\frac{|{A}_{L}-{A}_{U}|}{{A}_{L}}$$

## Results and discussion

### Characterization of fGO

**Figure 2:**
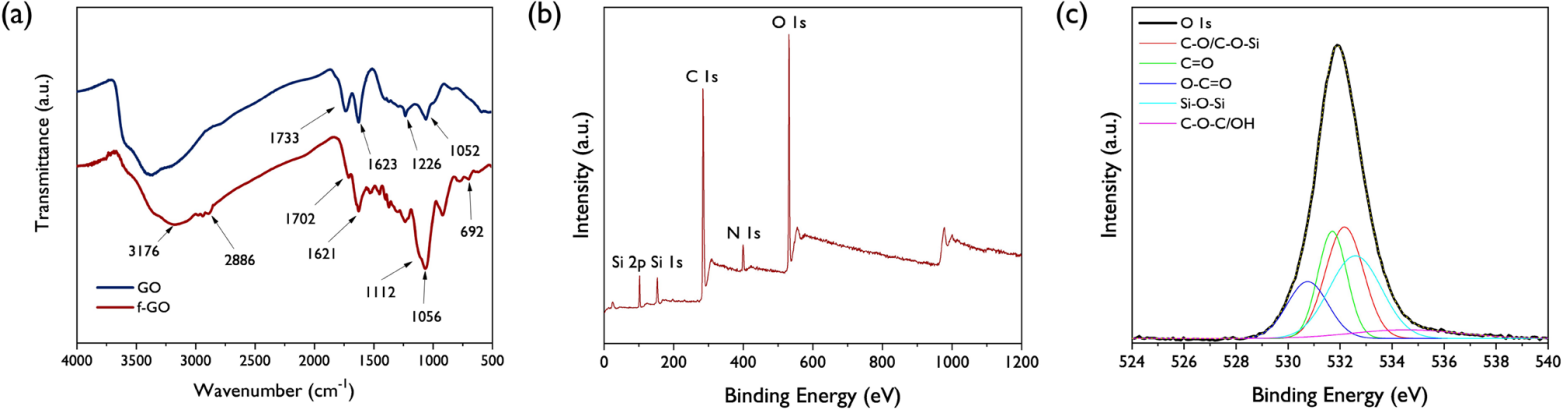
(**a**) FT-IR spectra of the GO and fGO, (**b**) overall XPS spectra of fGO, and O 1 s spectrum (**c**).

**Figure 3:**
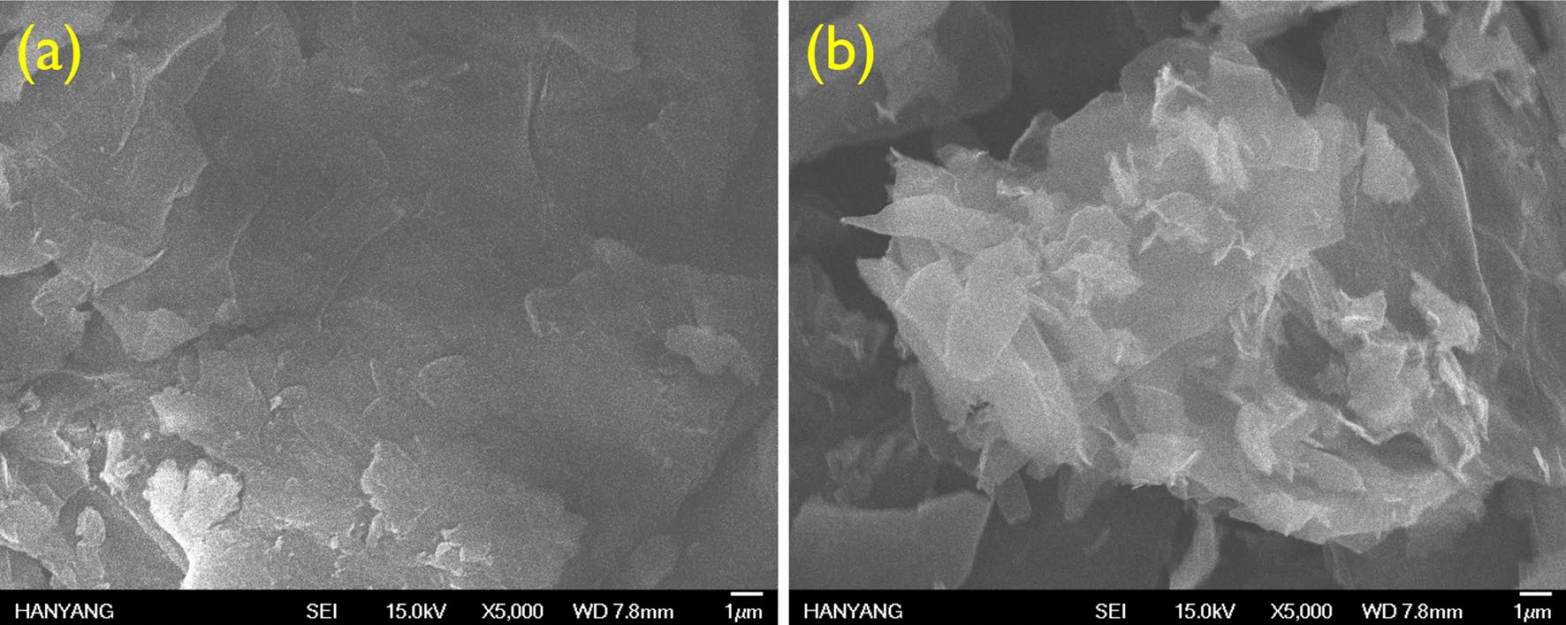
FE-SEM images of (**a**) GO and (**b**) fGO.

In this report, fGOs were prepared by grafting APTES on GO. GO exfoliated by strong oxidation of graphite contains various functional groups, including epoxy (1,2-ether, not 1,3-ether) groups as a main functional group, and some hydroxyl, carbonyl, and carboxyl groups present at the edge. Based on many studies, silane materials grafted on GO have been reported such as 3-methacryloxypropyltrimethoxysilane (MPMS), gamma-(2,3-epoxypropoxy) propyltrimethoxysilane (EPMS), phenyltriethoxysilane (PES), triethoxyvinylsilane (VES), 3-glycidoxypropyltrimethoxysilane (GPTS), APTMS, and APTES^31−33^. The GO and silane materials reacted in two major pathways depending on variable reaction conditions such as temperature, pH, and the ratio of reactants. The first path was silanization formed by the covalent –Si–O–Si– bond, which originated from the alkoxy groups on APTES attacked and displaced by the hydroxyl groups of GO. The second path is the epoxy ring opening of GO with the amine groups present in APTES^34^. The two different reaction routes between GO and APTES occurs simultaneous (Scheme 1). To characterize the fGO-prepared structure, FT-IR and XPS analyses were performed and the results are depicted in Fig. 2. As shown in Fig. 2a, compared with the spectrum of GO, the newly formed absorption bands around 3176 cm^−1^ and 2886 cm^−1^ mean that the N–H stretching in amine and the C–H stretching in aliphatic CH_2_ groups, respectively. More clear evidence for the successful silanization can be confirmed by strong stretching vibrations for Si–O at 1056 cm^−1^, as well as the stretching and bending vibrations of Si–O–C at 1112 and 692 cm^−1^, respectively. The peaks at 102, 284, 399, and 531 eV in XPS analysis represent the Si 2p, C 1s, N 1s, and O 1s lines, respectively. Based on numerous studies, the XPS spectrum of pristine GO shows only two peaks, namely, C 1s and O 1s. Thus, the presence of the N 1s and Si 2p peaks of fGO confirm successful functionalization of the GO with APTES. In agreement with the FT-IR analysis, the O 1s signal splits into five components of O–C=O (530.2 eV), C=O (531.2 eV), C–O/C–O–Si (531.8 eV), Si–O–Si (532.7 eV), and C–O–C/OH (533.1 eV), which are also described in Fig. 2c^35^. The FE-SEM images of GO and fGO sheets are shown in Fig. 3. The pristine GO sheets show tightly stacked structure and smooth surface at the edge. It can be seen that the fGO exists as separated thin layers with rough surface at the edge. These results indicated that silane surface treatment effectively prevents GO stacking.

### Preparation of fGO–PU nanocomposites

The fGO–PU nanocomposites were prepared by in situ polymerization using CO as a triol crosslinker and fGO as a nanosized reinforcement. The important factors for successful polymerization of the fGO–PU nanocomposite are the concentration of reactants, reaction time, and reaction temperature. In particular, the concentration of reactants is the most critical factor for stable preparation of the fGO–PU nanocomposite. In the first step of the reaction, an NCO-terminated PU prepolymer was synthesized through a reaction between PCL-diol and excess HDI. In the second step, the desired amount of fGO and CO as a crosslinker were added dropwise at a highly diluted concentration in DMF. The final dilution of reactant in DMF was below 10 wt%.

### Characterization of fGO–PU nanocomposites

#### Structural analysis

**Figure 4:**
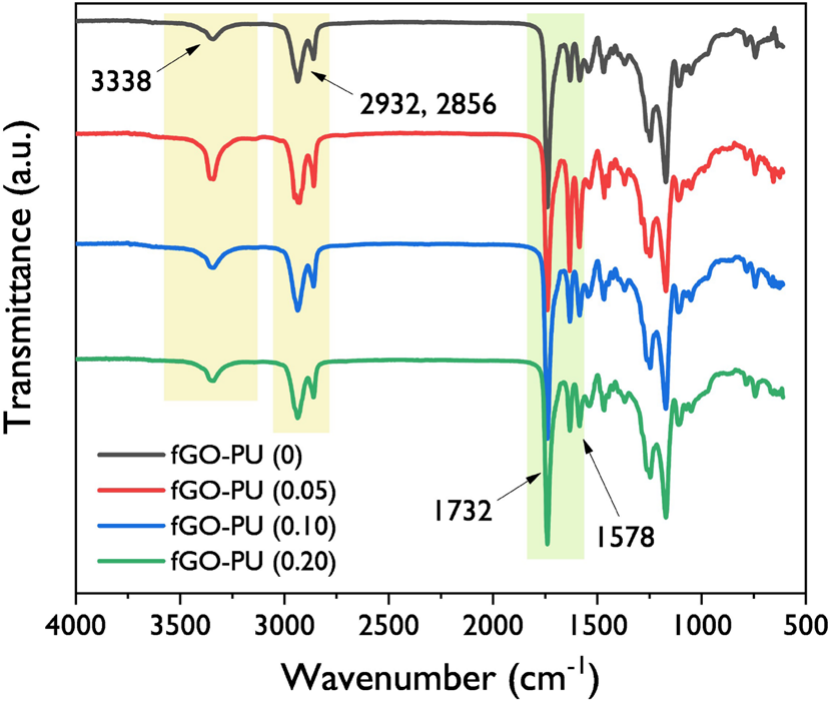
Overall FT-IR spectra of the fGO–PU 0, fGO–PU 0.05, fGO–PU 0.10, and fGO–PU 0.20.

**Figure 5:**
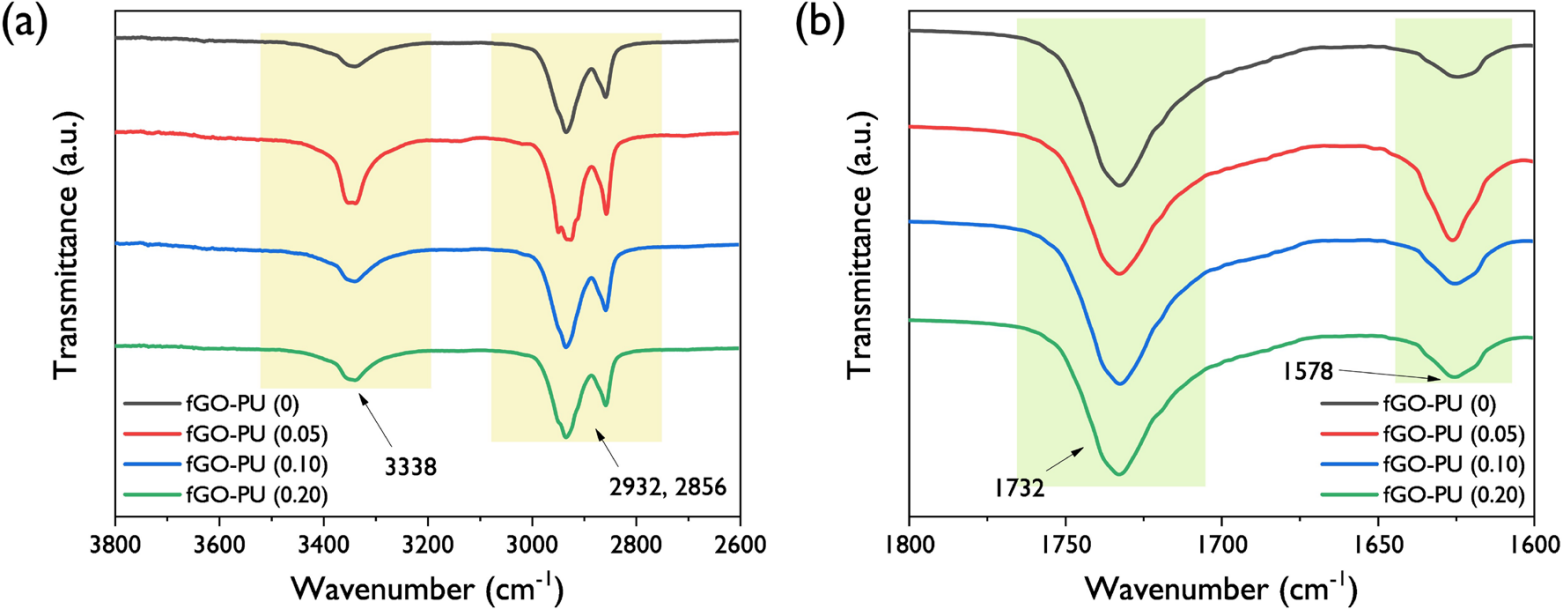
(**a**) The amplified > N–H absorption band and (**b**) > C=O absorption bands of fGO–PU 0, fGO–PU 0.05, fGO–PU 0.10, and fGO–PU 0.20.

FT-IR spectra of the fGO–PU nanocomposites are shown in Fig. 4. The disappearance of the isocyanate peak at 2265 cm^−1^ in all fGO–PU nanocomposites confirmed that the polymerization was completed successfully. Compared with the spectrum of PCL-diol, the newly formed absorption bands around 3338 and 1732 cm^−1^ mean that N–H stretching and C=O carbonyl stretching are present in the urethane linkage. The bands found at 2932 and 2856 cm^−1^ emerged due to symmetric *sp*^2^ and asymmetric *sp*^3^ stretching bands in the aliphatic chain of PCL-diol^36^. Another band at 1578 cm^−1^ corresponds to amide II governed by in-plane N–H bending and C–C stretching vibrations. It is worth noting the peaks were expected by incorporation of fGO, but no significant peaks were associated with fGO in the nanocomposites because only a very small amount of fGO was added and the bands were overshadowed by other bands^37^. Although it is difficult to find direct evidence for the fGO insertion, we can explore the changes in the structure of fGO–PU nanocomposites because FT-IR provides meaningful information on the extent and strength of hydrogen bonding present in PUs. According to Kumari et al*.*, the N–H stretching and C=O stretching bands are very sensitive to hydrogen bonding present in the PU structure because they act as proton donors and proton acceptors, respectively^38^. By studying the shift in frequency of the absorption band and the change in intensity of these bands, we could estimate the change in structures or the environment around the functional groups in PU. In general, the N–H and C=O stretching bands give information on hydrogen bonding in PU. According to Gunes et al*.*, in the case of PCL-diol-based PU it is hard to distinguish the hydrogen-bonded carbonyl peaks (1700 cm^−1^) from the free carbonyl absorption (1730 cm^−1^) present in PCL-diol^39^. As shown in Fig. 5b, in this case, the carbonyl peaks found in the range of 1750 cm^−1^ and 1700 cm^−1^ overlap. Thus, the N–H stretching gives more detailed information on hydrogen bonding caused by urethane linkage. In Fig. 5a the N–H band of fGO–PU 0.05 shows a sharper peak than fGO–PU 0.10 and fGO–PU 0.20. Therefore, the extent of hydrogen bonding caused by urethane linkage is higher than that of fGO–PU 0.10 and fGO–PU 0.20. This result is attributed to the presence of crosslinking, which hinders hydrogen bonding between N–H and C=O groups. With the increase in added fGO, more crosslinking points are formed, and there is more disturbance of hydrogen bonding. The fGO–PU nanocomposite in this study has an isocyanate-rich formula; the isocyanate and the amine groups in fGO can form urea linkages. Therefore, multiple hydrogen bonds can be formed with three types of proton donors such as the urethane N–H, the urea N–H, and the amide N–H groups, and four types of proton acceptors, namely, urethane C=O, urea C=O, amide C=O, and the C–O–C groups^40,41^. The various types of hydrogen bonding that arise from these cause numerous controversies in structure analysis. In this case, a crucial piece of information can be gained on the hydrogen bonding caused by urea linkage (see Fig. 5b, the region between 1650 and 1600 cm^−1^). The absorption band at 1625 cm^−1^ means a bidentate hydrogen bond with urea carbonyl. The band for fGO–PU 0.05 is larger than that of fGO–PU 0.10 and fGO–PU 0.20. It is suggested that when a relatively small moiety of fGO is loaded, the amount of urea linkage formed is small so that there is a high probability of forming a bidentate hydrogen bond with the neighboring urea linkages.

#### Crystallinity

**Figure 6:**
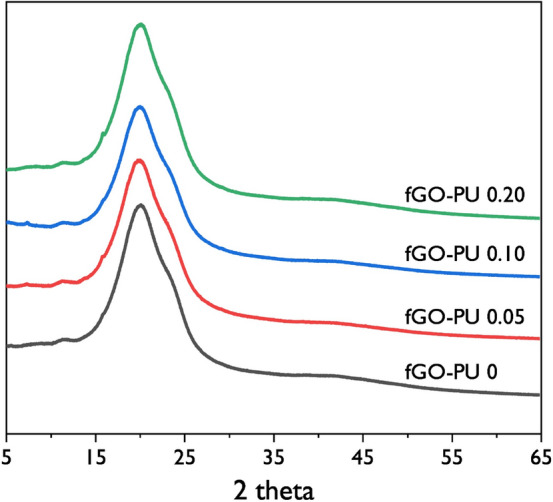
XRD patterns of fGO–PU 0, fGO–PU 0.05, fGO–PU 0.10, and fGO–PU 0.20.

The XRD patterns for the fGO–PU nanocomposites are shown in Fig. 6. It could be confirmed that the fGO–PU nanocomposites were basically amorphous because no distinctive peaks were observed except for the broad diffraction band at 2*θ* = 20.2°. In some PU studies using PCL-diol and CO, the characteristic peaks of PCL-diol were often observed; as well, the nucleating effects in PCL-diol were reported by introducing surface-modified GO as a filler. However, in this study, development in crystal structure was not observed, which is attributed to the dominant effect of crosslinking networks in formation by an excess of isocyanate groups. Crosslinked bonding in PU not only reduces the mobility of the hard and soft segments but also limits their ability to pack into crystalline phases^42^.

#### Morphology

**Figure 7:**
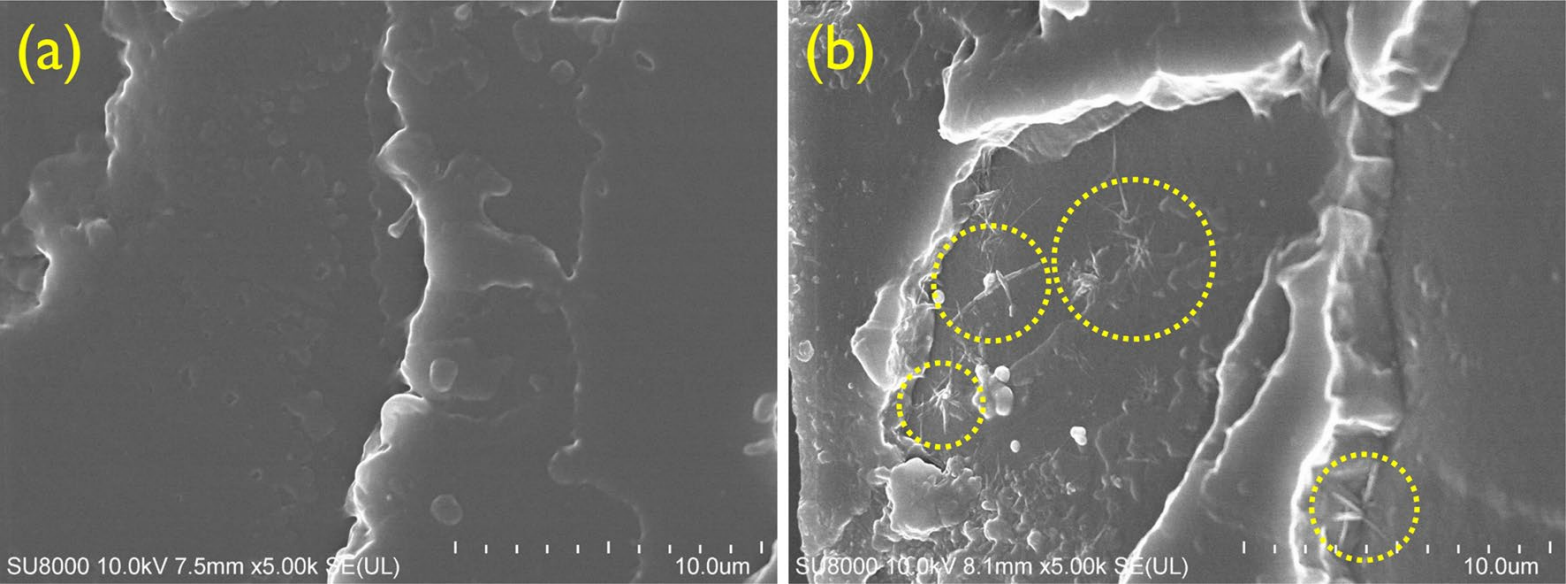
FE-SEM images of (**a**) fGO–PU 0 and (**b**) fGO–PU 0.20.

The morphological study for the fGO–PU nanocomposites was carried out using scanning electron microscopy (SEM). The SEM images of the samples taken at a magnification of 5000× are shown in Fig. 7. Compared with fGO–PU 0, as the fGO was loaded, a fuzzy interface was created between the fGO and the PU matrix. This indicates that a strong chemical urea bond (–NH–C=O–NH–) was formed between the amine group in the fGO and the excess NCO in the PU chains. For the fGO–PU 0.20, a star-shaped bonding structure radiating in all directions is observed, which plays a key role in enhancement in the mechanical and thermal properties to be described below.

#### DSC analysis

**Figure 8:**
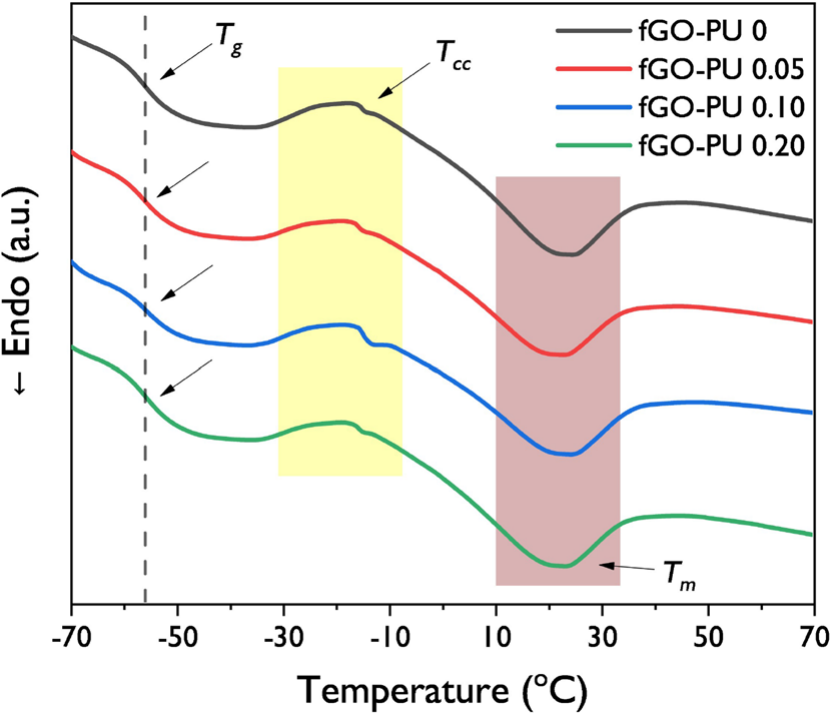
The DSC second heating scans of fGO–PU 0, fGO–PU 0.05, fGO–PU 0.10, and fGO–PU 0.20.

The thermal properties of fGO–PU nanocomposites were determined by DSC analysis. The DSC heating scans are shown in Fig. 8 and the thermal properties calculated from the second heating scans are listed in Table 2. All the fGO–PU samples show transition temperature (*T*_*g*_), crystallization temperature (*T*_*c*_), and melting temperature (*T*_*m*_) peaks. It is known that the PCL-diol shows no defective *T*_*g*_ peak in the DSC thermogram. In this case, the heating thermograms for the fGO–PU nanocomposites show an obvious inflection point where the baseline is not recovered. This means *T*_*g*_ can be observed as crosslinking points introduced in PCL-diol chains. However, there is no significant difference in transition temperatures regardless of fGO introduction. Meanwhile, an exothermic transition of the soft segment was observed, which corresponds to the crystallization peak during the heating cycle. These results are due to the recrystallization of the polycaprolactone soft segment. As listed in Table 2, with an increase in fGO content, the crystallization temperature in the heating scan (*T*_*c*_) shifts to a lower temperature and the heat of crystallization also decreases. The crosslinking caused by the introduction of fGO in PU interferes with the recrystallization in the soft segment, which leads to a decrease and broadening in crystallization temperature. Behera et al*.* reported that they have confirmed this phenomenon by comparing the PCL-diol-based linear PU with crosslinked PU using 1,4-butanediol as a chain extender and ionic triol as a crosslinker^36^.

**Table 2:** Thermal properties of fGO–PU nanocomposites.

Sample	*T*_*g*_ (°C)	*T*_*m*_ (°C)	*T*_*c*_^b^ (°C)	Δ*H*_*m*_^a^ (J g^−1^)	Δ*H*_*c*_^b^ (J g^−1^)
fGO–PU 0	− 56.6	22.1	− 16.7	11.1	6.1
fGO–PU 0.05	− 55.9	20.1	− 17.5	10.4	6.1
fGO–PU 0.10	− 56.1	21.9	− 17.6	11.5	5.6
fGO–PU 0.20	− 56.0	20.6	− 17.8	10.7	5.7

^a^Heat of fusion per gram of the corresponding polymer.

^b^Heat of crystallization per gram of the corresponding polymer in the heating cycle.

#### TGA

**Figure 9:**
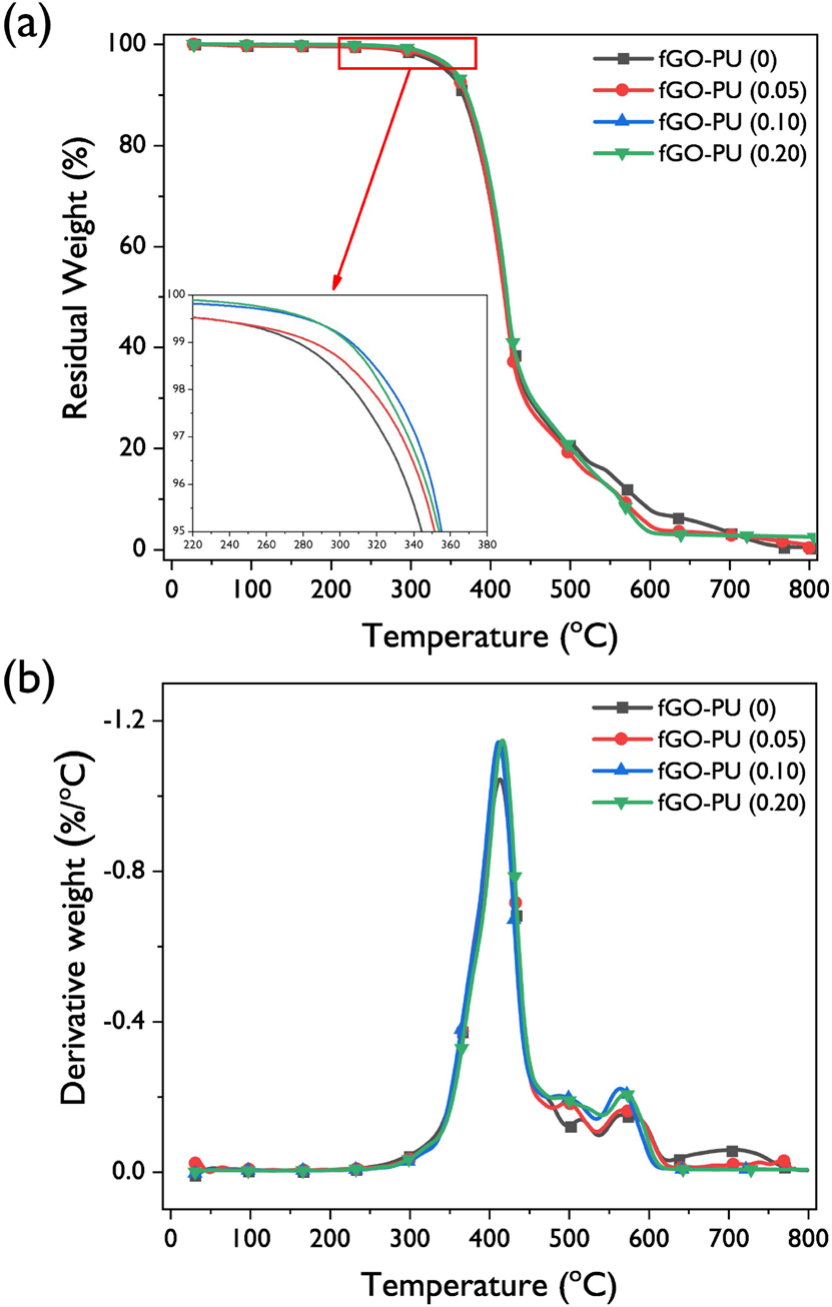
TGA thermograms of (**a**) fGO–PU 0, fGO–PU 0.05, fGO–PU 0.10, and fGO–PU 0.20 and (**b**) their derivatives.

To estimate the thermal stability of the fGO–PU nanocomposites, a TGA was carried out in a nitrogen atmosphere. TGA thermograms and their derivative curves are shown in Fig. 9. The initial decomposition temperatures for the fGO–PU nanocomposites (inset of Fig. 9a) were shifted to higher temperatures by incorporation of fGO. As shown in Fig. 9b, the fGO–PU nanocomposites show three thermal degradation stages. Based on many studies, the thermal degradation behavior of vegetable oil-based PU is represented by three steps. To further investigate the effect of the fGO on the thermal degradation behavior for the fGO–PU nanocomposites, a TGA kinetic analysis was performed for the three steps. The activation energy for thermal degradation (${E}_{a}$) of the fGO–PU nanocomposites can be estimated from the TGA thermograms using the Horowitz–Metzger kinetic method given below^43−46^:

$$\mathit{ln}\left[ln{\left(1-\alpha \right)}^{-1}\right]=\frac{{E}_{a}\theta }{R{T}_{dm}^{2}}\boldsymbol{^{\prime}}$$

where *α* is the fractional weight loss and *θ* is the variable auxiliary temperature (*θ* = *T* − *T*_*dm*_). *R* is the universal gas constant and *E*_*a*_ can be calculated from the slope of a linear fitting of $\mathit{ln}\left[ln{\left(1-\alpha \right)}^{-1}\right]$ versus *θ*. The obtained *E*_*a*_ values of the fGO–PU nanocomposites are listed in Table 3 for each step. In addition, the calculated *E*_*a*_ values exhibited good reliance for describing the thermal degradation kinetics of the fGO–PU nanocomposites by the fact that the coefficient of determination (*r*^2^) values were higher than 0.99. At the first step, the urethane bonds’ degradation starts at the urethane bond. The urethane bond is known to have a relatively low thermal stability. At this stage, three mechanisms of degradation of the urethane bond proceeded simultaneously. These are (1) dissociation to isocyanate and alcohol, (2) formation of primary amine and olefin, and (3) formation of secondary amine. In our case, the first phase proceeded up to about 480 °C and the maximum decomposition temperatures of all samples were listed below the first phase in Table 3. As a result, the maximum decomposition temperatures and *E*_*a*_ of the first step tended to increase with the incorporation of fGO. In particular, the *E*_*a*_ values increased from 127.0 kJ for fGO–PU to 136.9, 140.4, and 143.1 kJ for the fGO–PU 0.05, 0.10, and 0.20, respectively. This means that more energy is needed to break the urethane bond with fGO loading. At the second step, the maximum degradation temperatures and *E*_*a*_ were found to have similar values for all samples. This stage is related to the oligomerization of the triglyceride structure in CO. In our previous study, we reported the thermal decomposition behavior of PU prepared using CO-based multifunctional polyols and various diisocyanates. It has been confirmed that there is no significant effect on the second stage thermal decomposition behavior for vegetable oil-based PUs regardless of the type of polyol and diisocyanate used^12^. The final step is due to the complete degradation of the remainders of the second stage. At this step, the *E*_*a*_ tended to increase markedly according to the fGO content. As in the thermal decomposition behavior, the second step is indifferent, it can be assumed that the degradation of the grafted fGO occurs below this step.

**Table 3:** Thermal degradation parameters of fGO–PU nanocomposites.

Sample	*T*_*id*_ (°C)^*a*^	1st phase		2nd phase		3rd phase	
		*T*_*dm*_ (°C)^b^	*E*_*a*_ (kJ)	*T*_*dm*_ (°C)	*E*_*a*_ (kJ)	*T*_*dm*_ (°C)	*E*_*a*_ (kJ)
fGO–PU 0	306.9	414.5	127.0	516.2	26.0	568.4	22.8
fGO–PU 0.05	316.5	415.8	136.9	486.6	27.0	568.9	24.2
fGO–PU 0.15	327.8	412.7	140.4	488.7	28.4	565.5	46.6
fGO–PU 0.20	323.4	417.7	143.1	490.6	28.3	572.2	49.1

^a^Initial thermal degradation temperatures at 2% weight loss.

^b^Temperatures at maximum degradation rates.

#### Mechanical properties

**Figure 10:**
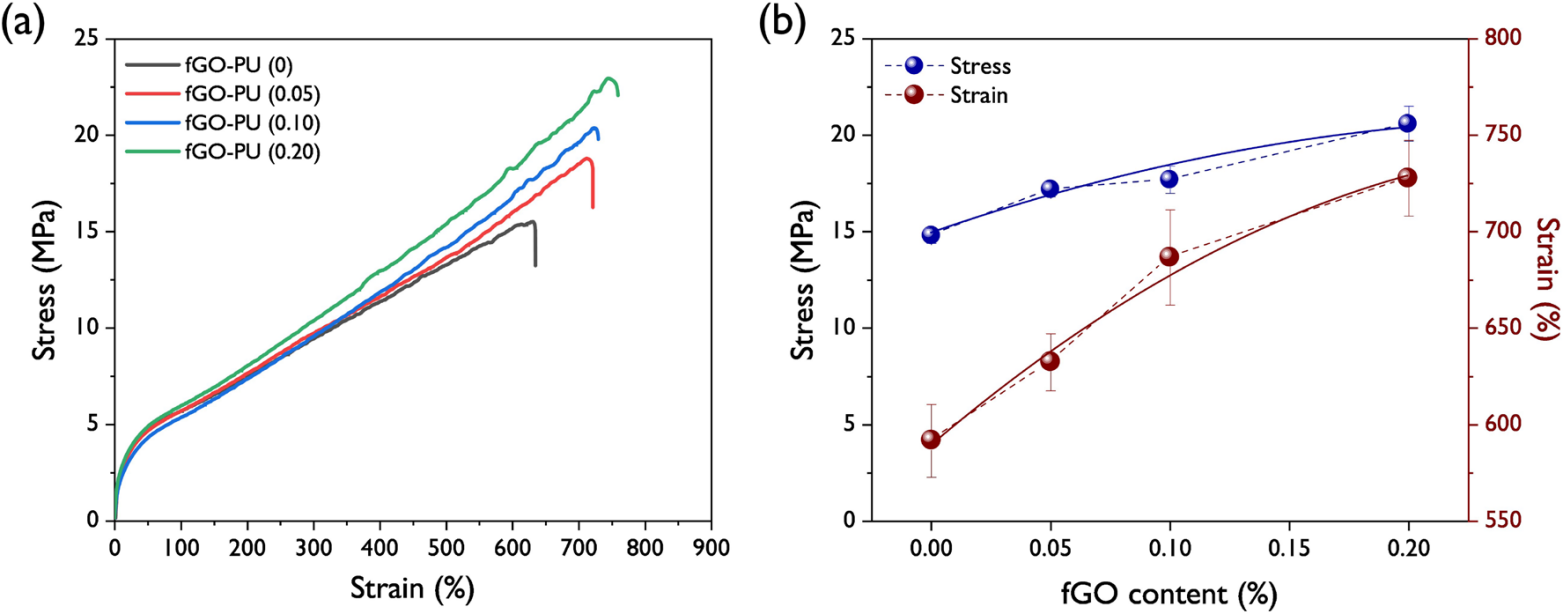
(**a**) Representative stress–strain profiles and (**b**) maximum stress and strain on fGO content.

The representative stress–strain curves of the fGO–PU nanocomposites are shown in Fig. 10 and their mechanical properties including tensile strength, elongation at break, and toughness are listed in Table 4. The fGO–PU 0 possesses low tensile strength, elongation, and toughness due to the monoglyceride group of CO. After loading the fGO, all the nanocomposites show excellent mechanical performance in terms of the abovementioned properties. The noteworthy achievements in this study are that incorporation of fGO leads to enhancement not only in tensile strength but the elongation at break of fGO–PU nanocomposites results in improvement in toughness. In particular, with the initial loading of fGO (0.05 wt%), significant improvements in mechanical properties are observed, compared with fGO–PU 0. This result can be explained by the formation of chemical crosslinks and hydrogen bonds that were derived from the reaction between the excess isocyanates in PU chains and the hydroxyl and amine groups present in fGO. In addition, further discussion is needed on the improvement in elongation accompanied by stress enhancement. This result is often reported in nanocomposites using graphene-based nanosheets, including GO, modified GO, and graphene nanoplatelets as a reinforcement. According to Thakur et al*.*, this phenomenon may be attributed to slippage of graphene-based fillers over each other in nanocomposites lying in a high tensile state^47^.

**Table 4:** Mechanical properties of fGO–PU nanocomposites.

Property	fGO–PU 0	fGO–PU 0.05	fGO–PU 0.10	fGO–PU 0.20
Tensile strength (MPa)	14.8 ± 0.4	17.2 ± 0.4	17.7 ± 0.7	20.6 ± 0.9
Elongation at break (%)	591.7 ± 18.9	632.4 ± 14.8	686.7 ± 24.7	727.8 ± 19.6
Toughness^a^ (MJ m^−3^)	54.4 ± 2.8	65.9 ± 3.4	70.5 ± 4.3	84.7 ± 4.8

^a^Calculated from stress–strain curves.

**Figure 11:**
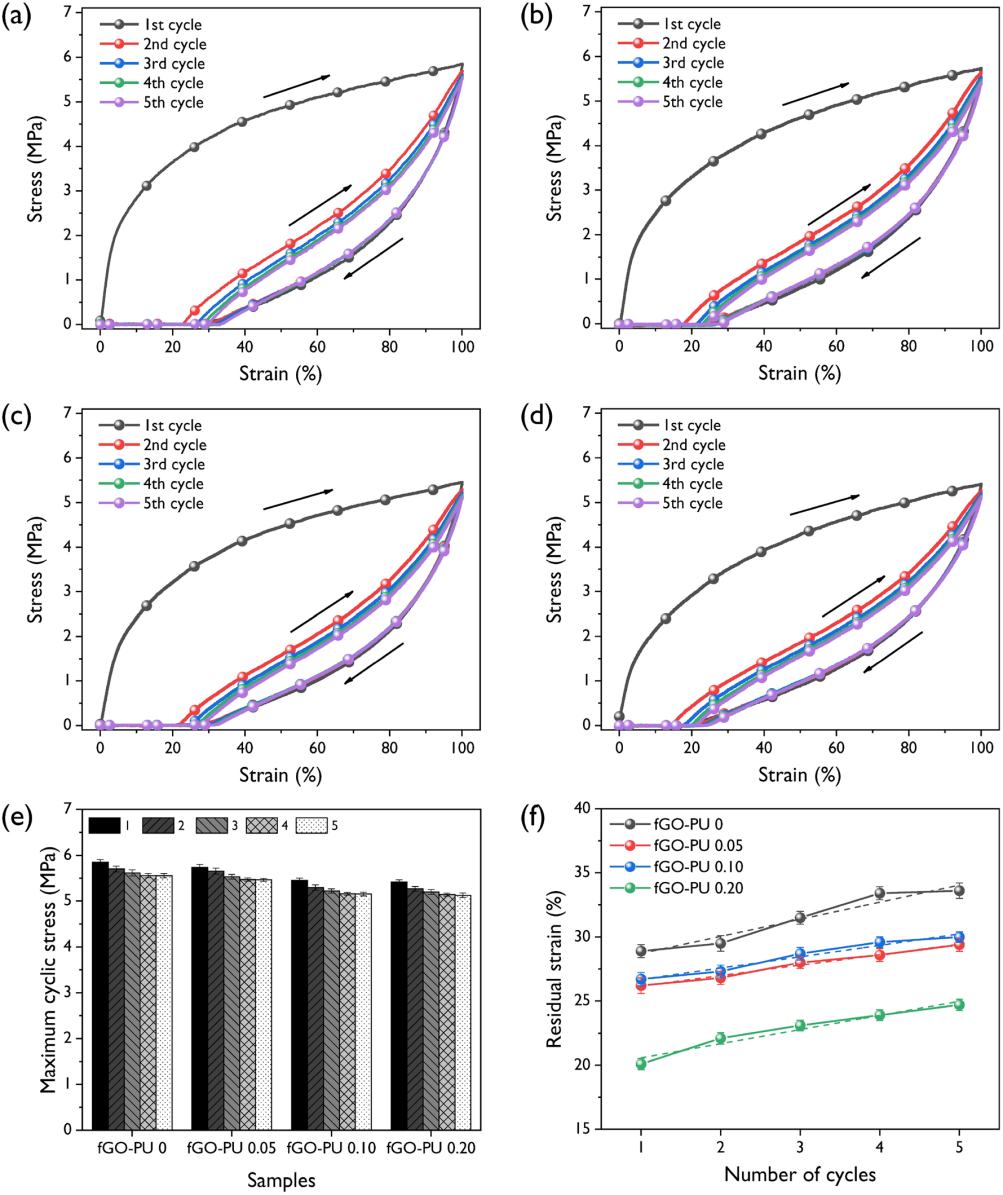
Stress–strain profiles of elastic recoveries at 100% for (**a**) fGO–PU 0, (**b**) fGO–PU 0.05, (**c**) fGO–PU 0.10, and (**d**) fGO–PU 0.20. (**e**)The changes in maximum cyclic stresses for the fGO–PUs according to recovery cycles with different fGO contents. (**f**) Mechanical hysteresis of fGO–PUs as a function of fGO content.

**Figure 12:**
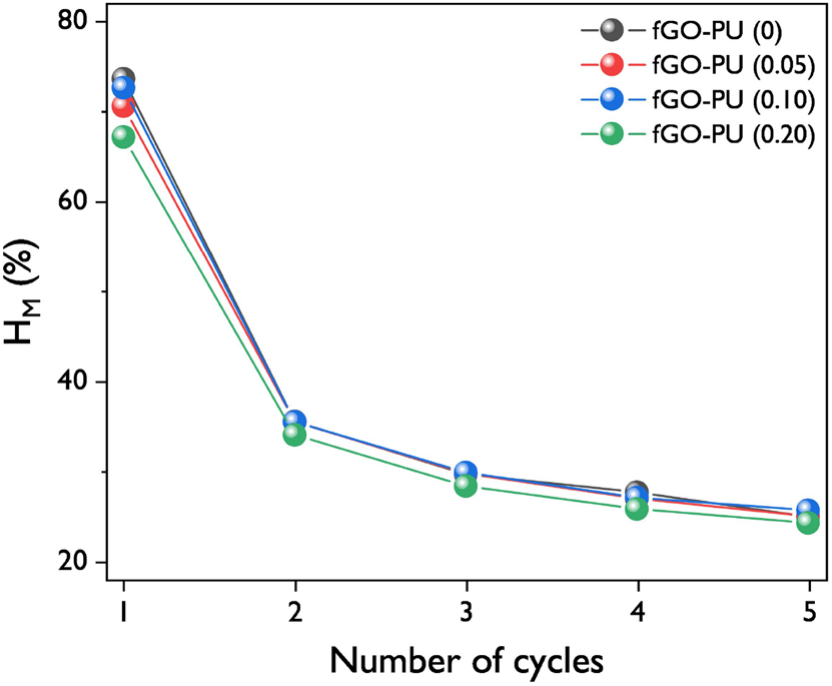
Mechanical hysteresis of fGO–PU nanocomposites as a function of fGO contents.

To evaluate the elastic recovery property of fGO–PU nanocomposites, a cyclic mechanical test was carried out and the results are shown in Fig. 11. As shown in Fig. 11a–d, there is a huge hysteresis curve between the first and second cycles for all fGO–PU samples. However, no significant difference was observed from the second to the fifth cycle. This is probably due to strong softening caused by chain orientation or rearrangement of hard segments in the first cycle. In another view of this, it may be caused by the deformation and failure in the structure of the nanocomposites, called “the special training effect” shown in the first cycle of a shape memory polymer. Figure 11e describes the change in maximum cyclic stresses of fGO–PU nanocomposites as a function of the cycle. At the first cycle, the cyclic stress rapidly decreased till the third cycle where all samples showed a relatively stable state. Strain recovery after the first cycle was also determined from the cyclic test and the results are summarized in Fig. 11f. As the cycles progressed, the strain recovery tended to increase for all samples. Moreover, with the loading of fGO, the strain recovery generally decreased from 29% (corresponding to a residual plastic deformation of 71%) for the pristine fGO–PU to 20% for the fGO–PU 0.20 in the first cycle. This may be caused by the fact that it is difficult to deform the structure due to crosslinking and hydrogen bonding in the fGO–PU nanocomposites^48^. This result can be explained by the hysteresis of fGO–PU nanocomposites determined from the area between the loading–unloading curves (Fig. 11a–d). To summarize the mechanical hysteresis (*H*_*M*_), the changes in *H*_*M*_ are presented in Fig. 12. As the fGO content increased, the values of *H*_*M*_ decreased. This phenomenon could be explained by an excellent chain interaction such as chemical crosslinking, hydrogen bonding, and compatibility at the PU matrix and fGO.

## Conclusion

fGO, which corresponds to APTES-grafted GO, was prepared and successfully characterized using FT-IR and XPS analysis. fGO–PU nanocomposites were produced in situ with different contents of fGO. From the FT-IR spectra, the chemical structures of fGO–PU nanocomposites were analyzed in detail focusing on the formation of crosslinks, urea linkages, and changes in hydrogen bonding in PU. Crystallinity, thermal properties, and thermal degradation behavior for all fGO–PU nanocomposites were investigated. The result of *E*_*a*_ kinetics in particular indicates that the incorporation of a very small amount of fGO into PU improved the thermal stability of the nanocomposites. All the fGO–PU nanocomposites showed enhanced mechanical properties, including tensile strength, elongation, toughness, and hysteresis with an increase in the fGO loading level.
